# Three-Dimensional Analysis of Ferrite Grains Recrystallized in Low-Carbon Steel during Annealing

**DOI:** 10.3390/ma14154154

**Published:** 2021-07-26

**Authors:** Kengo Horiuchi, Toshio Ogawa, Zhilei Wang, Yoshitaka Adachi

**Affiliations:** Department of Materials Design Innovation Engineering, Graduate School of Engineering, Nagoya University, Furo-cho, Chikusa-ku, Nagoya 464-8603, Japan; horiuchi.kengo@c.mbox.nagoya-u.ac.jp (K.H.); wang.zhilei@material.nagoya-u.ac.jp (Z.W.); adachi.yoshitaka@material.nagoya-u.ac.jp (Y.A.)

**Keywords:** recovery, recrystallization, carbon, three-dimensional analysis, Avrami exponent

## Abstract

We performed a three-dimensional (3D) analysis of ferrite grains recrystallized in low-carbon steel during annealing. Cold-rolled specimens were heated to 723 K and held for various periods. The 3D morphology of ferrite grains recrystallized during the annealing process was investigated. The progress of recovery in low-carbon steel was more inhibited than that in pure iron. However, ferrite recrystallization in low-carbon steel was more rapid than that in pure iron. The Avrami exponent was inconsistent with the 3D morphology of the recrystallized ferrite grains in pure iron but consistent with that of the grains in low-carbon steel. Thus, the Avrami exponent depends on the recovery and recrystallization behaviors. Furthermore, the recrystallized ferrite grain growth was virtually 2D. Three types of recrystallized ferrite grains were observed: recrystallized ferrite grains elongated along the transverse or rolling direction; plate-shaped recrystallized ferrite grains grown in the transverse and rolling directions; fine and equiaxed recrystallized ferrite grains. These results suggest that the recrystallized ferrite grains did not grow in the normal direction. Thus, we concluded that the 3D morphology of recrystallized ferrite grains depends on the kinetics of recrystallization and the initial microstructure before recrystallization.

## 1. Introduction

Low-carbon steels are widely used for automobile parts due to their high strength and excellent formability [1]. Controlling metallurgical phenomena in manufacturing processes, including recrystallization, phase transformation, and precipitation, is vital to obtain excellent materials properties. We focused on controlling ferrite recrystallization in low-carbon steels during annealing [2,3,4,5,6,7].

In our previous studies, we reported remarkable retardation in ferrite recrystallization when steel is heated above the ferrite-to-austenite transformation temperature before the completion of ferrite recrystallization [2]. Moreover, we demonstrated that recrystallized ferrite grains are equiaxed and the distribution of martensite after annealing is homogeneous when the initial microstructure includes martensite [6]. These previous studies have shown that ferrite recrystallization plays an essential role in controlling the microstructure of low-carbon steel.

In addition, we have examined the three-dimensional (3D) morphology of ferrite grains recrystallized during annealing [8]. The Johnson–Mehl–Avrami–Kolmogorov (JMAK) theory [9,10,11] was applied to various materials for the 3D analysis of recrystallization. The JMAK equation is expressed as follows:

*X* = 1 − *exp* (−*kt^n^*) (1)

where *X* is the fraction recrystallized, *k* is a constant, *t* is time, and *n* is the Avrami exponent. The Avrami exponent in Equation (1) and the 3D morphology of recrystallized grains are correlated [12]. The Avrami exponent is estimated to be between 1 and 2, 2 and 3, or 3 and 4 if the morphology of the recrystallized grains is 1D, 2D, or 3D, respectively. Some previous studies [13,14,15,16] reported that the Avrami exponent might not reflect the 3D morphology of the recrystallized grains. In the case of pure iron, we found that the inconsistency between the Avrami exponent and 3D morphology of recrystallized grains is attributed to the remarkable decrease in the driving force for the ferrite recrystallization at the later stage of annealing due to the rapid progress of recovery at the early stage of annealing [8].

The presence of solute carbon retards recovery in iron and steel [17,18,19], meaning that the progress of recovery in low-carbon steel is more retarded than that in pure iron. If the recovery progress is retarded by solute carbon in low-carbon steel, the Avrami exponent may correspond with the 3D morphology of recrystallized grains. This study investigated the relationship between the Avrami exponent and the 3D morphology of recrystallized ferrite grains. Additionally, we characterized the 3D morphology of recrystallized ferrite grains and attempted to suggest the mechanism of 3D microstructural evolution at the ferrite recrystallization process.

## 2. Materials and Methods

Fe-0.11 mass% C steel without other alloying elements was used herein. Vacuum-melted ingot was hot-rolled in the austenite region, and the hot-rolled sheet was cold-rolled at a cold reduction rate of 80% (thickness: 0.8 mm). Afterward, the cold-rolled specimens were heated (MILA-5000, ULVAC, Kanagawa, Japan) to 723 K at a heating rate of 10 K·s^−1^, held for various periods at that temperature, and then water-quenched to room temperature (298 K ± 2 K).

Vickers hardness tests (HMV-1, Shimadzu, Kyoto, Japan) were carried out under an applied load of 9.8 N for 10 s to quantify the fraction recrystallized, *F_R_*, using Equation (2) [6]:

(2) $$F_{R}=\frac{H_{NR}-H_{W}}{H_{NR}-H_{R}}\;\times \;100$$

where *H_NR_* and *H_R_* are the Vickers hardness of the specimen cold-rolled and completely recrystallized, respectively; *H_w_* is the Vickers hardness of the entire specimen. The standard deviation was calculated from the results obtained from three specimens. *F_R_* is an index of fraction softened. However, it is used as an index of the recrystallized fraction. The decrease in *H_w_* is mainly attributed to the progress of ferrite recrystallization. In Equation (2), *F_R_* increases with a decrease in *H_w_* because *H_NR_* and *H_R_* are constant. For instance, if *H_w_* equals *H_NR_*, (immediately before the start of ferrite recrystallization), *F_R_* is 0%, and if *H_w_* equals *H_R_*, (immediately after the completion of ferrite recrystallization), *F_R_* is 100%.

The microstructure of nital-etched specimens was observed using an optical microscope (BX51, Olympus, Tokyo, Japan). The recrystallized ferrite grain size was measured using the linear intercept method (ASTM E112-13 [20]). A microtexture analysis at the quarter-thickness position in the rolling-direction–normal-direction (RD–ND) plane was performed using an electron backscatter diffraction/field emission scanning electron microscopy (EBSD/FEGSEM, JSM-7001FA, JEOL, Tokyo, Japan) system employing orientation imaging microscopy (OIM) software (step size: 0.2 μm, version 7.3.1, TSL solutions, Kanagawa, Japan). The microstructures (250 × 300 μm^2^) were observed with 28 sections and a total depth of approximately 23 μm using a fully-automated serial sectioning 3D microscope (Genus_3D, Nakayamadenki, Osaka, Japan) [21]. Moreover, the 3D recrystallized ferrite grains were built from the serial-sectioned images using Amira software (version 6.5.0, Maxnet, Tokyo, Japan), and the length (maximum Feret diameter), breadth (maximum horizontal measurement taken at right angles to the length), and width (minimum Feret diameter) of the 3D recrystallized ferrite grains could be obtained.

We evaluated the dislocation density by X-ray diffraction (XRD) line-profile analysis, called the modified Williamson–Hall and Warren–Averbach methods. Diffraction angle *θ* and integral breadth *β* could be obtained in the X-ray diffraction peaks, and dislocation density could be estimated using these parameters. Details were reported in our previous article [22]. The XRD patterns of the specimens were obtained using an X-ray diffractometer (Ultima IV, Rigaku, Tokyo, Japan) with Cu Kα radiation of wavelength *λ* = 0.15418 nm at 40 kV and 40 mA (scanning speed: 0.1° min^−1^). The standard deviation was calculated from the results obtained for at least two specimens.

## 3. Results

**Figure 1:**
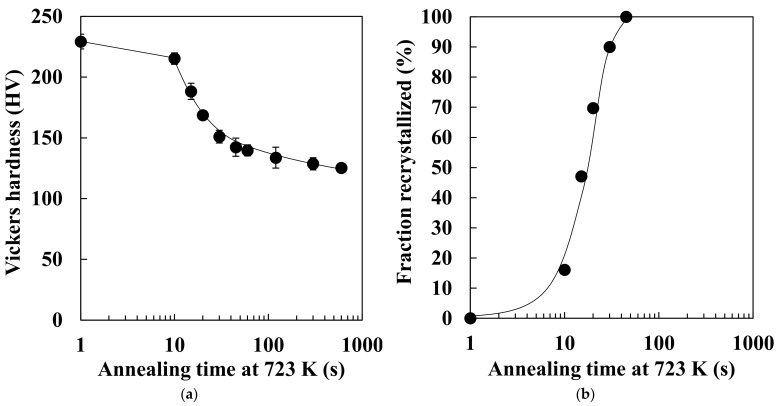
Changes in (**a**) Vickers hardness and (**b**) recrystallized fraction during isothermal holding at 723 K.

**Figure 2:**
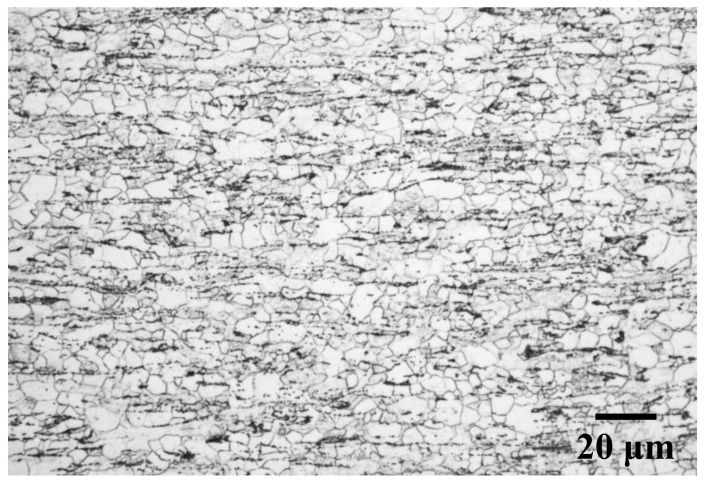
Microstructure of specimens annealed at 723 K for 45 s.

The changes in Vickers hardness and recrystallized fraction during annealing at 723 K are shown in Figure 1. The hardness slightly decreased at the early stage of annealing (up to 10 s), and then drastic softening was observed immediately after the start of ferrite recrystallization. Therefore, recovery likely occurred until the isothermal holding time reached 10 s. Figure 2 displays the microstructure of specimens annealed at 723 K for 45 s. White and black areas in the figure correspond to recrystallized ferrite grains and cementite particles, respectively. Ferrite recrystallization was completed when the isothermal holding time reached 45 s because nonrecrystallized ferrite grains were observed, and the average size of recrystallized ferrite grains was approximately 4.8 μm.

**Figure 3:**
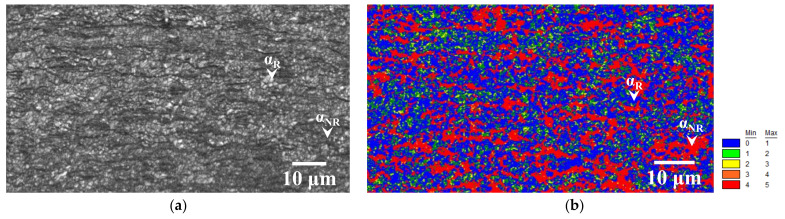
(**a**) Image quality and (**b**) kernel average misorientation maps of specimens annealed at 723 K for 15 s (*α_R_*: recrystallized ferrite grain; *α_NR_*: nonrecrystallized ferrite grain).

Image quality (IQ) and kernel average misorientation (KAM) maps of the specimen annealed at 723 K for 15 s are presented in Figure 3, where very fine recrystallized ferrite grains were observed (Figure 3a). The region with KAM lower than 1° corresponds to recrystallized ferrite grains [2]. Additionally, the recrystallized ferrite grain fraction evaluated by KAM analysis and the Vickers hardness tests were almost the same (approximately 47%). Moreover, KAM values increase with an increase in dislocation density (red regions in Figure 3b) [2,23], suggesting that nonrecrystallized ferrite grains have a higher dislocation density than recrystallized ferrite grains. The changes in dislocation densities during the recovery process evaluated by the modified Williamson–Hall and Warren–Averbach methods, are listed in Table 1. The dislocation density in pure iron remarkably decreased when annealed at 723 K for 10 s [22]. In the case of low-carbon steel, the dislocation densities in the cold-rolled and 10-s-annealed specimens were almost the same (approximately 1.7 × 10^15^ m^−2^).

**Figure 4:**
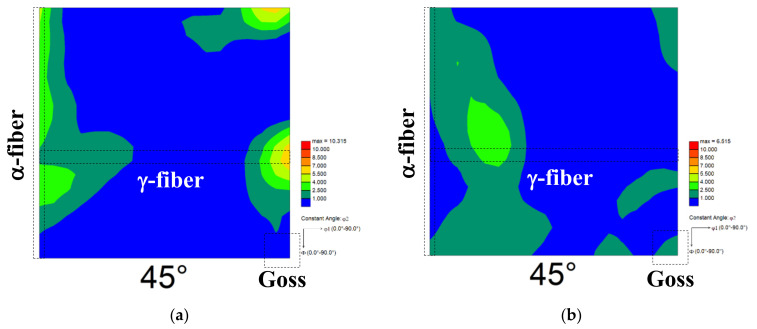
Global texture for specimens annealed at 723 K for (**a**) 10 s and (**b**) 15 s (φ2 = 45° section).

Figure 4 illustrates the global texture of the specimen annealed at 723 K for 10 and 15 s, respectively. As shown in Figure 4a, α-fiber (RD//<110>) and γ-fiber (ND//{111}) were primarily observed at the early stage of annealing. In addition, Goss orientation ({110}<001>) was observed when the isothermal holding time reached 15 s (Figure 4b).

**Figure 5:**
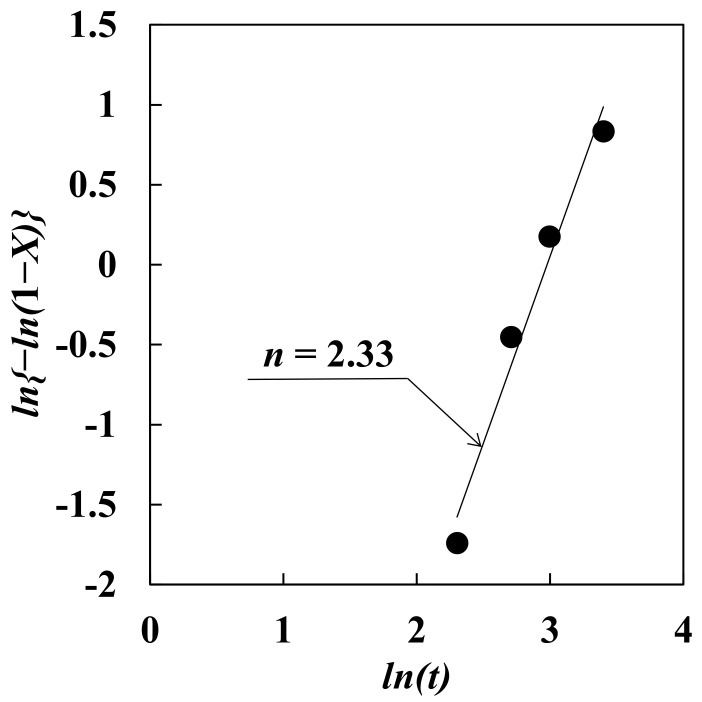
Johnson–Mehl–Avrami–Kolmogorov plot of recrystallization kinetics at 723 K.

Figure 5 depicts the JMAK plot of the recrystallization kinetics at 723 K. Rearranging Equation (1), we obtain the following:

*ln*{−*ln*(1 − *X*)} = *nln*(*t*) + *B* (3)

where *B* is a constant. Equation (3) signifies that the slope of Figure 5 is the Avrami exponent; the Avrami exponent was computed as 2.33. As mentioned previously, the Avrami exponent is estimated to be between 1 and 2, 2 and 3, or 3 and 4 if the morphology of the recrystallized grains is 1D, 2D, or 3D, respectively. Thus, the morphology of the recrystallized ferrite grains is probably close to 2D.

**Figure 6:**
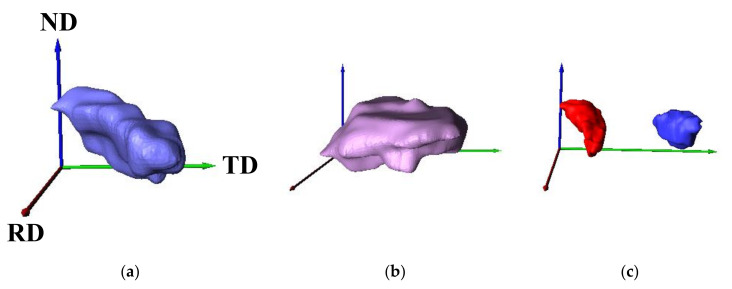
Typical 3D recrystallized ferrite grains (**a**) elongated in the transverse or rolling direction, (**b**) grown in the transverse and rolling directions, and (**c**) fine and equiaxed in specimens annealed at 723 K for 45 s (ND: normal direction; TD: transverse direction; RD: rolling direction).

Figure 6 exhibits the 3D recrystallized ferrite grains in the specimen annealed at 723 K for 45 s. There were three types of recrystallized ferrite grains:

(1) Recrystallized ferrite grains elongated along the transverse or rolling direction (Figure 6a).
(2) Plate-shaped recrystallized ferrite grains grown in the transverse and rolling directions (Figure 6b).
(3) Fine and equiaxed recrystallized ferrite grains (Figure 6c).

**Figure 7:**
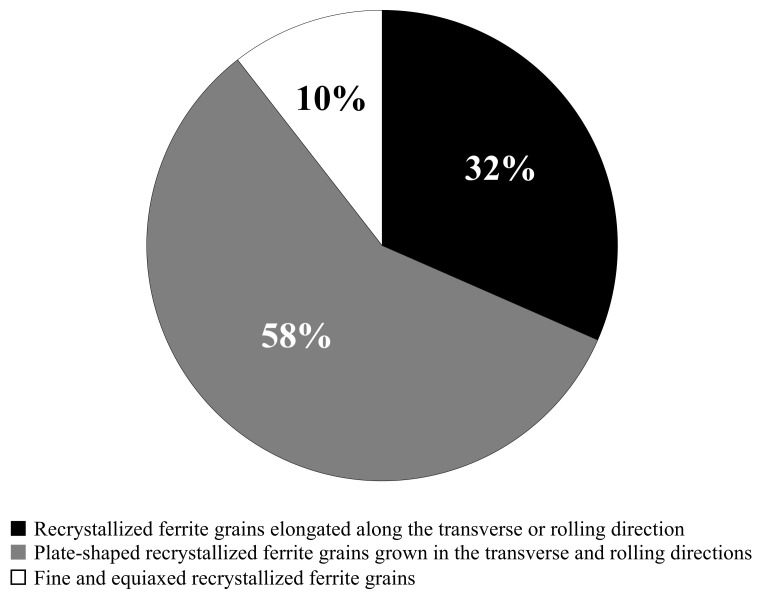
Fraction of 3D recrystallized ferrite grain morphology.

Furthermore, a fraction of each 3D recrystallized ferrite grain is presented in Figure 7. Most of the recrystallized ferrite grains grew in the transverse and/or rolling direction.

**Figure 8:**
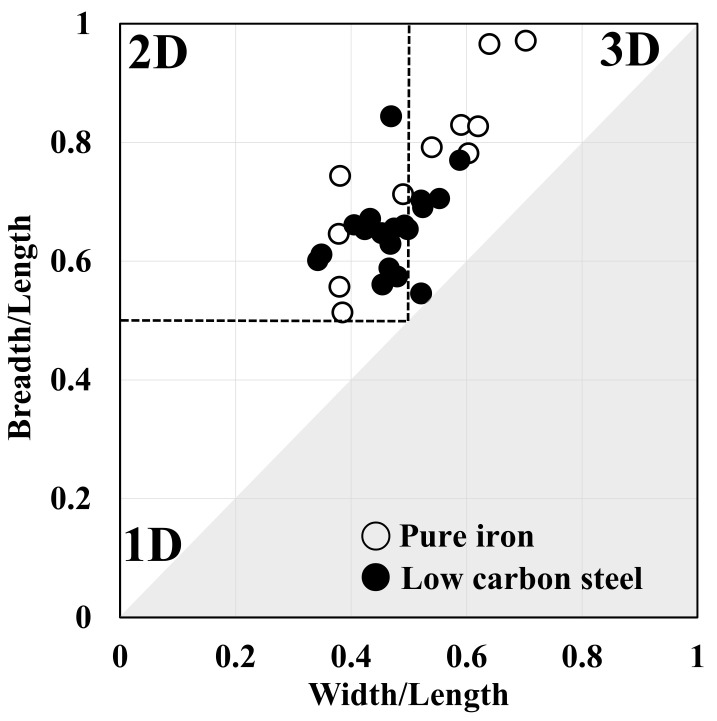
Quantitative evaluation of 3D recrystallized ferrite grains (Data of pure iron was taken from a previous report [8]).

Figure 8 displays a quantitative evaluation of the 3D recrystallized ferrite grains in the specimens annealed at 723 K for 45 s. We assumed that the morphology of the recrystallized ferrite grains is close to 1D if breadth/length and width/length ratios are less than 0.5. We assumed that the morphology of the recrystallized ferrite grains is approximately 3D if the breadth/length and width/length ratios are more than 0.5. In addition, we presumed that the morphology of the recrystallized ferrite grains is practically 2D if the breadth/length ratio was more than 0.5 and the width/length ratio was less than 0.5. As shown in Figure 8, the morphology of recrystallized ferrite grains was nearly 2D. Figure 5 and Figure 8 reveal that the Avrami exponent is consistent with the 3D morphology of recrystallized ferrite grains.

## 4. Discussion

A major finding in this study is that the Avrami exponent is consistent with the 3D morphology of recrystallized ferrite grains in low-carbon steel. In the case of pure iron, we reported that the Avrami exponent is inconsistent with the 3D morphology of recrystallized ferrite grains [8]. The inconsistency is attributed to the remarkable decrease in the driving force for the ferrite recrystallization at the later stage of annealing due to the high recovery rate at the early stage. These outcomes imply that the recovery process plays an important role in the 3D analysis of recrystallized ferrite grains. Here, we discuss the recovery and recrystallization behaviors during annealing and the 3D morphology of the recrystallized ferrite grains based on previous reports.

### 4.1. Recovery and Recrystallization Behaviors

As shown in Figure 1 and Table 1, the hardness and dislocation density were unchanged immediately before the start of ferrite recrystallization in low-carbon steel. Moreover, the nonrecrystallized ferrite grains that remained after the start of ferrite recrystallization exhibited high dislocation density (Figure 3). In contrast, subgrains were formed before ferrite recrystallization in pure iron [8]. These results indicate that recovery in low-carbon steel was significantly more inhibited than in pure iron. Solute carbon retards recovery in iron and steel [17,18,19], consistent with the results obtained in this study.

Furthermore, the ferrite recrystallization duration in low-carbon steel annealed at 723 K was less than that of pure iron annealed at 823 K [8], signifying that the ferrite recrystallization is more rapid in low-carbon steel than in pure iron. The decrease in dislocation density during the recovery process was extraordinary in pure iron, whereas it was insignificant in low-carbon steel. The driving force for recrystallization is expressed as the following equation [3]:

*G* = 0.5 × *ρμb*^2^ (4)

where *G* is the driving force for recrystallization, *ρ* is the dislocation density, *μ* is the shear modulus, and *b* is the Burger’s vector. Assuming *μ* is 80 GPa and *b* is 2.5 × 10^−10^ m, the driving force for recrystallization could be calculated by instituting the dislocation density listed in Table 1 into Equation (4). As a result, the driving forces for recrystallization in pure iron and low-carbon steel annealed at 723 K for 10 s were calculated to be 9.3 × 10^5^ and 4.4 × 10^6^ J·m^−3^, respectively. Therefore, one of the reasons for the difference in ferrite recrystallization behavior is that the driving force for ferrite recrystallization is higher in low-carbon steel than in pure iron. In addition, ferrite recrystallization is accelerated by solute carbon because several new recrystallized ferrite grains are generated in the deformation bands of steel containing a trace amount of solute carbon [24]. It was reported that recrystallized ferrite grains with the Goss orientation are preferentially nucleated in the deformation bands [25]. As shown in Figure 4, the Goss orientation ({110}<001>) was developed, which is attributed to the formation of deformation bands during cold-rolling. This result implies the formation of deformation bands during cold-rolling.

Based on our comparison, the results obtained herein establish that recovery was more inhibited in low-carbon steel than in pure iron, whereas ferrite recrystallization was more rapid in low-carbon steel than pure iron.

### 4.2. Three-Dimensional Analysis

As shown in Figure 8, the morphology of recrystallized ferrite grains in low-carbon steel was approximately 2D. Moreover, the recrystallized ferrite grains grown in the transverse and/or rolling direction were mainly observed immediately after the completion of ferrite recrystallization (Figure 6 and Figure 7). These outcomes indicate that the recrystallized ferrite grains barely grew in the normal direction.

**Figure 9:**
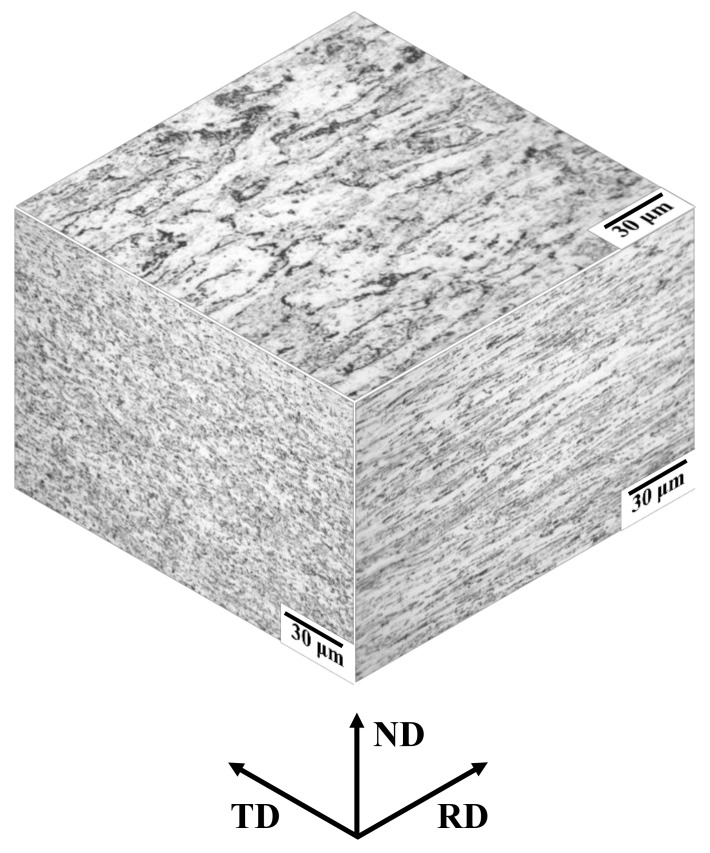
Three-dimensional microstructure of as-cold-rolled specimen.

Recrystallized ferrite grains hardly migrate across nonrecrystallized ferrite grain boundaries, although the misorientation angles between recrystallized and nonrecrystallized ferrite grains are large enough for ferrite recrystallization [26]. Herein, the 3D microstructure of the as-cold-rolled specimen is displayed in Figure 9. The spacing between high-angle grain boundaries was larger in the order of rolling (61.1 ± 22.7 μm), transverse (7.50 ± 2.64 μm), and normal (3.16 ± 1.02 μm) directions. Moreover, the spacing between high-angle grain boundaries in the normal direction of the cold-rolled specimen is close to the average size of fully recrystallized ferrite grains. Thus, it is likely that recrystallized ferrite grains hardly grew in the normal direction because the spacing between high-angle grain boundaries in the normal direction is relatively small. As a result, it appears that recrystallized ferrite grains grew preferentially in the transverse and/or rolling directions.

In the case of pure iron, subgrains are formed before ferrite recrystallization, and the recrystallized ferrite grains are comparatively equiaxed in three dimensions immediately after completion of ferrite recrystallization [8]. In contrast, the recovery was not observed immediately after the start of ferrite recrystallization in low-carbon steel, as presented in Figure 1 and Table 1. This implies that continuous recrystallization, such as subgrain growth, occurs in pure iron, and the kinetics of recrystallization in pure iron and low-carbon steel vary. It was reported that the 2D morphology of recrystallized ferrite grains depends on the recovery process, and the progress of ferrite recrystallization due to subgrain growth results in comparatively equiaxed ferrite grains [19]. Accordingly, the 3D morphology of recrystallized ferrite grains may also depend on the kinetics of recrystallization. In addition, the spacing between high-angle grain boundaries in the normal direction in pure iron (24.9 ± 6.1 μm) [8] is larger than that of low-carbon steel (3.16 ± 1.02 μm). Thus, recrystallized ferrite grains in pure iron could grow more in the normal direction than those in low-carbon steel. In the case of low-carbon steel, fine and equiaxed recrystallized ferrite grains were observed (Figure 6c), and those grains may correspond to the ferrite grains recrystallized immediately after nucleation. Therefore, equiaxed recrystallized ferrite grains are likely to be observed if the size of the recrystallized ferrite grains is smaller than the spacing between high-angle grain boundaries in the normal direction.

**Figure 10:**
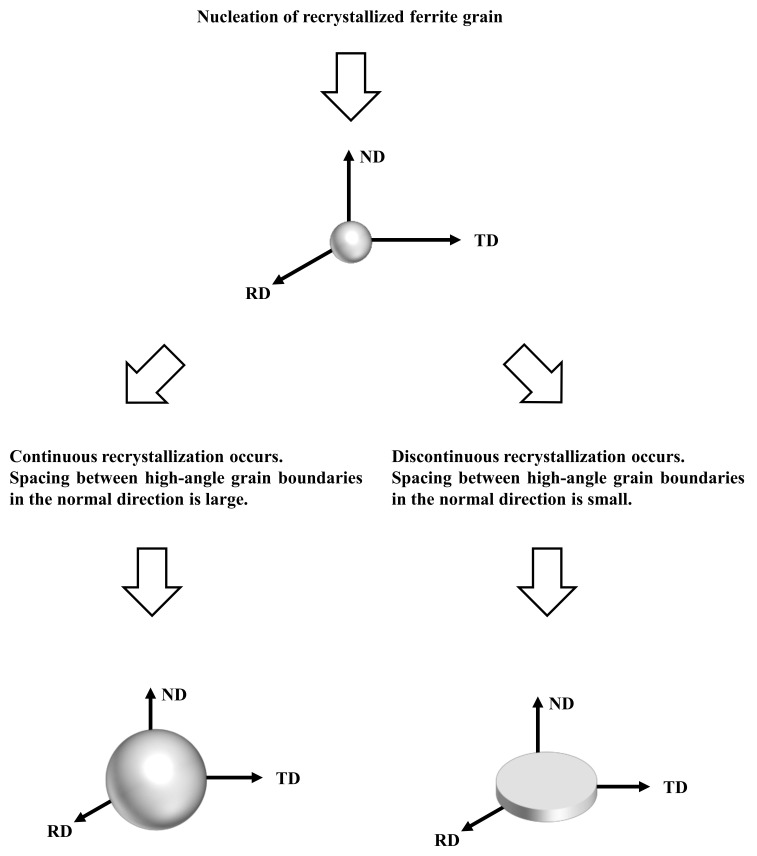
Schematics of 3D microstructural evolution during ferrite recrystallization.

The results obtained herein suggest that the 3D morphology of recrystallized ferrite grains depends on the kinetics of recrystallization and the initial microstructure before ferrite recrystallization. Figure 10 illustrates the 3D microstructural evolution during the ferrite recrystallization process. The recrystallized ferrite grains are comparatively equiaxed in three dimensions in the nucleation stage, irrespective of the material type. Furthermore, the recrystallized ferrite grain morphology becomes comparatively 3D when ferrite recrystallization due to subgrain growth (i.e., continuous recrystallization) occurs, and the spacing between high-angle grain boundaries in the normal direction is large. This finding is consistent with the results of pure iron in our previous report [8]. In contrast, the morphology of recrystallized ferrite grains becomes comparatively 2D when discontinuous recrystallization occurs, and the spacing between high-angle grain boundaries in the normal direction is small, which corresponds to low-carbon steel. In other cases, the morphology of recrystallized ferrite grains can become both 2D and 3D. For instance, the morphology of recrystallized ferrite grains can become 3D when the spacing between high-angle grain boundaries in the normal direction is large, even if discontinuous recrystallization occurs. On the other hand, the morphology of recrystallized ferrite grains can become 2D when the spacing between high-angle grain boundaries in the normal direction is small, even with continuous recrystallization occurs.

Three-dimensional morphology of various ferrite, such as Widmanstätten [27], acicular [28,29], allotriomorphic [30,31], and degenerate ferrite [32], were investigated, whereas that of simple polygonal ferrite has not been reported. Herein, we propose a model of the 3D evolution of recrystallized ferrite grains during the recrystallization process. However, the effect of texture on the 3D morphology of recrystallized ferrite grains was not considered. Furthermore, the obtained results indicate that the initial microstructure and cold reduction rate can affect the 3D morphology of recrystallized ferrite grains. These findings should be further investigated in the future.

## 5. Conclusions

We performed 3D analyses of ferrite grains recrystallized in low-carbon steel during annealing, and the following results were obtained.

(1) Recovery was more retarded in low-carbon steel than in pure iron, whereas ferrite recrystallization was more rapid in low-carbon steel than in pure iron.
(2) The Avrami exponent (*n* = 2.33) is consistent with the 3D morphology of recrystallized ferrite grains in low-carbon steel. Furthermore, a major portion of the recrystallized ferrite grains grew in the transverse and/or rolling directions.
(3) The 3D morphology of the recrystallized ferrite grains was dependent on the kinetics of recrystallization and the initial microstructure before recrystallization.

## Figures and Tables

**Table 1:** Dislocation densities of cold-rolled and 10-s-annealed specimens.

Materials	As-Cold-Rolled Specimen	10-s-Annealed Specimen
Pure iron (Ref. [22])	1.33 × 10^15^	3.70 × 10^14^
Low-carbon steel	1.64 × 10^15^	1.77 × 10^15^

## Data Availability

The data presented in this study are available on request from the corresponding author.
